# Understanding the Germicidal Effects of Silver Nanoparticles

**DOI:** 10.1289/ehp.120-a386

**Published:** 2012-10-01

**Authors:** Carol Potera

**Affiliations:** Carol Potera, based in Montana, has written for *EHP* since 1996. She also writes for *Microbe*, *Genetic Engineering News*, and the *American Journal of Nursing*.

Silver nanoparticles are an effective tool for killing disease-causing bacteria. But despite their widespread use in catheters, clothing, toys, cosmetics, and many other products,^1^ investigators haven’t fully understood whether their effectiveness is a function of the release of germicidal silver ions, some feature specific to their nanoparticle form, or both. Researchers at Rice University now report evidence that the release of dissolved silver ions is the driving force behind silver nanoparticles’ germicidal action.^2^

Silver ions are powerful antimicrobials, but they are easily sequestered by chloride, phosphate, proteins, and other cellular components.^3^ “Silver nanoparticles are less susceptible to being intercepted and a more effective delivery mechanism,” says Pedro J.J. Alvarez, chairman of Rice’s Civil and Environmental Engineering Department. The nanoparticle form is therefore used to ferry silver ions to bacteria they could not reach on their own, for example, by coating devices such as catheters. Silver ions are released from the particles in the presence of oxygen.^3^

In the new study, Alvarez and colleagues prevented oxidation by working inside a sealed chamber with no oxygen exposure. This allowed them to separate the roles played by silver ions and intact silver nanoparticles in killing *Escherichia coli*.

The researchers tested several types of silver nanoparticles found in commercial products as well as silver ions supplied as the salt AgNO_3_.^2^ They found that AgNO_3_ killed *E. coli* in a dose-dependent manner at concentrations as low as 15 μg/L. In contrast, silver nanoparticles did not kill *E. coli* even at concentrations up to 7,665 times higher than the minimum lethal concentration of silver ions themselves.

**Figure:**
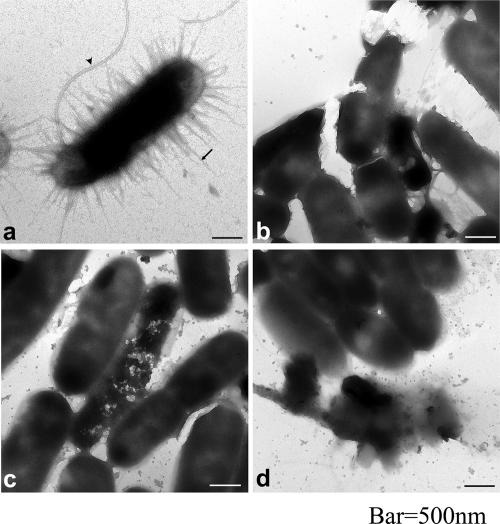
Nanoparticles are effective at delivering silver ions (shown here destroying E. coli^8^), but their nano nature does not appear to imbue them with additional antimicrobial properties. Jung et al.; doi:10.1128/AEM.02001-07

Surprisingly, concentrations of silver ions lower than 15 μg/L appeared to boost bacterial growth instead of killing it.^2^ According to Alvarez, this stimulatory response at low concentrations resembles suboptimal treatment with antibiotics, which can create resistant microbes. This observation suggests that “we may have to be careful not to undertreat with silver nanoparticles,” says Alvarez.

On the other hand, he adds, “We don’t want to overtreat either, because silver is an expensive disinfectant and causes collateral environmental damage.” Engineered silver nanoparticles have been detected in waterways, and laboratory studies have shown they can be toxic to higher organisms including ryegrass,^4^ algae,^5^ the nematode *Caenorhabditis elegans*,^6^ and fathead minnows,^7^ in addition to their potential effects on native microbial populations.

“This is a well-designed and -executed study of one of the more important questions about silver nanoparticles,” says Samuel N. Luoma, a research ecologist at the University of California, Davis. He says Alvarez’s findings support the continued use of silver nanoparticles.

The authors suggest the release of silver ions may be controlled through means such as better-designed polymer coatings. Ideally, silver could be released fast enough to kill bacteria yet slowly enough to avoid excess toxicity to the environment. “We need to be careful about nanoparticles but not afraid of them,” Alvarez says. “It’s good for the environment, business, and society that we are cautious.”
